# Retraction: Loss of FOXN3 in colon cancer activates beta-catenin/TCF signaling and promotes the growth and migration of cancer cells

**DOI:** 10.18632/oncotarget.28832

**Published:** 2026-04-17

**Authors:** Yuedi Dai, Meixing Wang, Haixia Wu, Mi Xiao, Houbao Liu, Dexiang Zhang

**Affiliations:** ^1^Department of Medical Oncology, Cancer Hospital of Fudan University, Minhang Branch, Shanghai 200240, China; ^2^General Surgery Department, General Surgery Institute, Zhongshan Hospital, Fudan University, Shanghai 200032, China; ^3^General Surgery Department, The Fifth People’s Hospital of Shanghai, Fudan University, Shanghai 200240, China; ^*^These authors have contributed equally to this work

**This article has been retracted:** Oncotarget has concluded its investigation into this paper following a retraction request from the corresponding author. The reason cited for the request was the non-reproducibility of data due to the use of incorrect cell lines. STR profiling revealed that the SW620 and SW480 cells used in the study were misidentified; they were actually HeLa and Huh7 (liver cancer) cells, respectively.

Additionally, our investigation identified several image duplications:

- Figure 2D: The “pcDNA3.1” soft agar assay panel overlaps with the Figure 3E “si FOXN3 1#” panel.
- Figure 2C: The “SW480/pcDNA3.1” motility assay panel overlaps with the Figure 3D “SW480/si con” panel.
- Figure 3D: The “SW620/si FOXN3 1#” motility assay panel is a duplicate of the “SW620/si FOXN3 2#” panel.
- Figure 6A: The “mouse 1” image in the “SW480-Luci/si con/49 days” panel was reused in an unrelated paper [1].

The use of incorrect cell lines and multiple instances of image duplication compromise the reliability of the experimental results. Consequently, the editorial decision has been made to retract this article. The authors have been notified of this decision.

Original article: Oncotarget. 2017; 8:9783–9793. 9783-9793. https://doi.org/10.18632/oncotarget.14189
